# Hyperspectral Imaging Study of Wheat Grains Infected with Several Fusarium Fungal Species and Their Identification with PCA-Based Approach

**DOI:** 10.3390/molecules30122586

**Published:** 2025-06-13

**Authors:** Anastasia Povolotckaia, Dmitrii Pankin, Mikhail Gareev, Dmitrii Serebrjakov, Anatoliy Gulyaev, Evgenii Borisov, Andrey Boyko, Sergey Borzenko, Sergey Belousov, Oleg Noy, Maxim Moskovskiy

**Affiliations:** 1 Federal Scientific Agroengineering Center VIM, 109428 Moscow, Russia; 2 Center for Optical and Laser Materials Research, St. Petersburg State University, 198504 St. Petersburg, Russia; dmitrii.pankin@spbu.ru (D.P.);; 3 Polytechnic Institute, Yaroslav-the-Wise Novgorod State University, 173000 Veliky Novgorod, Russia; 4 Don State Technical University, 346780 Rostov-on-Don, Russia; 5 Department of Processes and Machines in Agribusiness, Kuban State Agrarian University Named after I.T. Trubilin, 350044 Krasnodar, Russia; sergey_belousov_87@mail.ru; 6 L.L.C. OOO “Agroglobal Telecom”, 344012 Rostov-on-Don, Russia; oleg_neu@mail.ru

**Keywords:** wheat, *Fusarium*, hyperspectral imaging, reflectance, feature wavelength

## Abstract

Wheat is an important agricultural crop grown under various conditions on five continents. The ability to promptly detect and defeat fungal diseases has a significant impact on the volume of the obtained harvest. One of the most significant threats to human and domestic animal health is metabolites produced by *Fusarium* genus fungi. In this regard, this work is devoted to the possibility of the rapid differentiation between healthy grains and grains simultaneously infected with several species of *Fusarium* genus fungi (*Fusarium graminearum* Schwabe FG-30, *Fusarium poae* Kr-20-14, *Fusarium roseum* (*sambucinum*) St-20-3) for practical reasons. An approach based on obtaining hyperspectral data with their subsequent processing using the principal component analysis (PCA) method and determining statistically important spectral regions sensitive for grain infection at different stages (5 and 40 days) was proposed. The effects of the grain orientation and data dimensionality on the classification result were studied. For further practical application in devices for the rapid identification of wheat grains infected with *Fusarium*, a method based on the use of reflection at wavelengths of 400, 451, 708, 783, 801, and 863 nm, together with normalization [0, 1] and the subsequent projection of spectral data onto the first three principal components (PCs), was proposed, regardless of the grain orientation.

## 1. Introduction

Wheat is an important agricultural crop grown under various conditions on five continents [1]. It has been widely used since ancient times due to its high nutritional value and ease of processing and storage [1,2,3]. According to forecasts for the 2024–2025 trading year, 793 million metric tons of wheat are expected to be grown worldwide [4]. Fungal diseases can have significant impacts on the volume and quality of grown grains. The control of fungal contamination is primarily necessary to prevent the entry of hazardous-to-health toxic fungal metabolites into human and domestic animal food [5,6]. In this regard, an important task in practice is to detect contamination at the earliest stages during field work and under storage conditions of crops in grain storage facilities, as well as to control the degree of soil contamination [5,7,8].

In this regard, there is a need to develop methods for the monitoring and timely identification of infected grains for their subsequent separation. The currently existing methods can be conditionally divided into two large classes: field control methods and indoor control methods. The former approaches involve monitoring the condition of grains both in a single ear [9,10,11] and remote monitoring of sown areas, including using an unmanned aerial vehicle (UAV) [5,12,13,14,15,16,17]. Field methods have a number of their own features related to the influence of the environment, the movement of research objects, as well as certain technical requirements for devices, including weight, dimensions, power consumption, vibration resistance, etc.

At the same time, for devices located indoors, technical conditions unachievable for field devices to monitor the condition of a large volume of samples can be created. In this case, it becomes possible to analyze large volumes of samples, which is especially important when conducting in-line studies. However, the speed and difficulties in carrying out the analysis begin to act as a significant factor in choosing the analytical methods that are used as a basis.

Considering wheat, a significant disease affecting the volume of the obtained harvest and the quality of the grown grains is fusarium head blight—*Fusarium* genus fungal infection. It is one of the most common fungal diseases and poses the main threat to wheat [18]. During the development of this disease, various fungi produce dangerous metabolites, including secondary metabolites related to trichothecene family mycotoxins, such as T-2 and HT-2 toxin, as well as deoxynivalenol (DON) and zearalenone (Zea). Accumulating in the bodies of humans and animals, these toxins pose a significant threat to health, and their maximum concentrations are strictly controlled at the level of standards and regulations within countries, as well as at the global level [19].

Conventional laboratory methods for studying this fungal disease, such as chromatographic (HPLC and TLC) and PCR analysis methods, are complex to organize, destructive, and require highly pure consumables and control of experimental conditions, and the interpretation of the results requires appropriate employee qualifications [20,21,22,23,24,25]. At the same time, the microscopy method associated with growing and studying morphology is also very time-consuming and demands biological purity as well [24,26].

An alternative is optical analysis methods that allow the structure/state–spectrum correlation to be established both experimentally [22,24,27,28,29,30,31,32,33,34] and by modeling the individual metabolites (e.g., pigments and toxins) via modern theoretical methods [35,36,37,38,39,40,41,42,43]. These include methods related to the vibrational properties of the substances under study, for example, Raman spectroscopy [22,24,27,28] and IR absorption [24,29,44]. Their advantages include the relative ease of implementation, the possibility of non-destructive and contactless implementation for individual methods, as well as in some cases, the possibility of interpreting the obtained result by correlating it with certain bonds in the substance [24]. The most significant disadvantage is the locality of their application, which leads to the need for mapping.

Another type of method is associated with the optical properties arising from the electronic structures of materials, namely, reflection/absorption and luminescence [9,10,11,12,13,14,15,16,17,25,33,34,45,46,47,48]. And, if the latter is related to a specific composition, i.e., the content of a sufficient amount of highly luminescent pigments, for example, chlorophyll α [45,46], then the methods based on reflection and absorption are quite general, which allows them to be used to solve a wide range of problems. Despite the lower information content of the methods associated with complex electronic processes of optical radiation interaction with matter, methods based on reflection are promising in terms of their practical application in solving the problems of analyzing large amounts of information in a non-destructive, contactless manner. In this regard, the reflection phenomenon is used to obtain information in widely used methods such as the reflectance spectroscopy technique, hyperspectral imaging, and RGB imaging [5,9,10,11,12,13,14,15,16,17,49]. There are two main cases of spectral ranges for which information is obtained: the visible and partially near-IR (NIR) range (400–1000 nm) and the NIR range (mainly within 1000–2600 nm, i.e., SWIR region) [5]. This is, on the one hand, due to the type of detectors (Si or InGaAs type), and, on the other hand, due to the spectral features observed in these ranges. Thus, in the region of 1000–2500 nm, significant overtones and combination tones with hydrogen stretching vibrations of O-H, N-H, and C-H are noted [50]. They are distinguishable but already difficult to interpret up to 700 nm. While in the range of 400–800 nm, the manifestation of electronic absorption of pigments is noted [51].

The significant development in silicon detector fabrications, their relatively low cost in contrast to SWIR detectors [47], and high sensitivity make the range of 400–1000 nm attractive for practical application. Additional advantages in practice are the possibility of visual control in the visible range (380–700 nm), as well as a fairly sharp change in the reflection spectra of wheat grains in the range of 680–740 nm.

One of the currently relevant tasks is the possibility of separating infected and healthy grains based on reflectance spectral data in the range of 400–1000 nm [27,47,51,52]. In this case, reflectance data are obtained in hundreds of spectral bands, which are redundant. In this regard, these studies use data dimensionality reduction methods to identify the most significant spectral bands for subsequent classification by them. At the same time, an additional feature of the work [51] is the study of the possibility of a classification taking into account two grain orientations, the ventral and dorsal sides, with apparently one degree of infection. Additionally, in other works [27,47], the possibility of classifying grains with two degrees of infection was studied. Moreover, the best classification accuracy in the works [27,51,52] was 96.4%, 98%, 92% (overall), and 97%, respectively.

To date, currently, no studies have been presented that investigate the possibility of detecting infected and uninfected (healthy) grains simultaneously at certain infection times corresponding to the variants of early and long-term infections with the allocation of important spectral ranges and individual bands in this case. Additionally, it should be noted that in real conditions, an infection may be not with one type of fungus but with several at once, which requires a separate study of the synergistic effects of several *Fusarium* genus fungi [39]. Moreover, in reality, grains can also be oriented in an arbitrary way, which is more consistent with the situation of the location of an arbitrary grain on a conveyor belt.

Due to the fact that in real routine conditions the conveyor belt can move, the task of finding the minimum set of spectral bands important for classification will contribute to the minimum time of obtaining spectral data. Together with a simple and reliable classification algorithm, this will lead to the rapid processing of a large array of data.

In this regard, the task of this work was to study the possibility of detecting infected and healthy grains with an arbitrary orientation at different stages of infection, early (5 days) and late (40 days), based on spectral data from the range of 400–1000 nm. In this case, the test grains were infected with a selected set of several widely spread *Fusarium* fungal species from the infected grains of the 2024 harvest already studied in the laboratory. The task was also devoted to identify statistically valid ranges and feature wavelengths sensitive to the presence of infection, as well as to reduce the dimensionality of the data, while maintaining a sufficiently high level of classification accuracy. The obtained data and the classification accuracy will be discussed in combination with the previous conclusions made in former works. The demonstrated results may be of practical interest for the subsequent implementation of in-line devices and/or systems for a sufficiently fast analysis of a large number of grains.

## 2. Results and Discussion

### 2.1. Raman Spectroscopy Study

**Figure 1:**
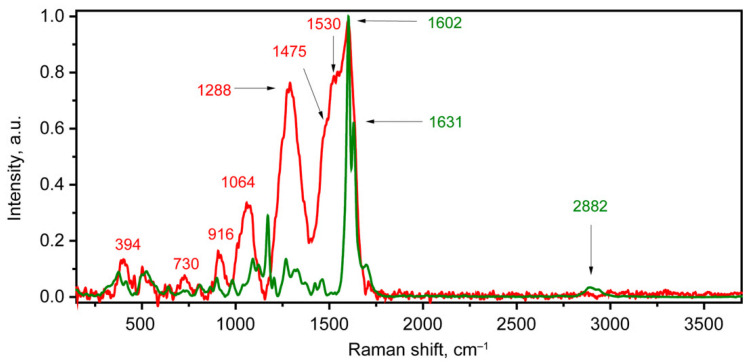
Examples of Raman spectra from the grains infected for 40 days (red) and healthy grains (green).

A significant difference between the spectra of grains infected for a long time and healthy grains was noted. Strong luminescence with a wide contour band of infected grains was manifested. An example of the Raman spectrum after subtracting the line terms and normalization is shown in Figure 1 (red curve). Two wide bands with maxima near 1600 and 1288 cm^−1^ can be distinguished. The band at 1600 cm^−1^ has a complex contour. Its maximum position is in the region of double carbon bonds. The contour of this band has a sharp decline on the side of large wavenumbers and an extension on the side of smaller wavenumbers with poorly resolved bands on the low-frequency side. A similar contour was noted in [24]. The positions of these poorly resolved peaks are around 1530 and 1475 cm^−1^. The frequencies of the peaks at 1530 and 1475 cm^−1^ are in the regions typical of deformational hydrogen vibrations with NH and CH bonds, respectively. Another significant band is the band around 1288 cm^−1^. Peaks close to the aforementioned frequency were noticed in a number of previous studies [53,54,55]. The 1288 cm^−1^ frequency is within the range of single bonds of C-C, C-O, and C-N, as well as the Amide III peak in the α helices of proteins [53,56]. Summarizing, based on the manifestation of the 1530 and 1300 cm^−1^ peaks, which may be related to Amide II and Amide III, respectively, as well as the broad contribution from the region around 1600 cm^−1^, a protein contribution to the spectrum could be concluded. While peaks at 730, 916, and 1064 cm^−1^ were attributed to stretching vibrations in C-C and C-O bonds, and the peak at 394 cm^−1^ was attributed to deformation vibrations in the carbon backbone of saccharides [54,56]. The Raman spectra obtained from the healthy grains look qualitatively different (Figure 1, green curve). In its high-frequency region, there is a clear presence of peaks from stretching vibrations in carbon–hydrogen bonds, identified by a band with a maximum at about 2882 cm^−1^. In the region below 1900 cm^−1^, the presence of an intense narrow doublet of bands around 1602 and 1631 cm^−1^ is noted, attributed to stretching vibrations in C=C bonds in aromatic rings [57,58]. A much lower relative intensity is shown by deformation vibrations involving hydrogen in the 1250–1475 cm^−1^ range, stretching vibrations including atomic displacements in single C-C and C-O bonds in the 750–1260 cm^−1^ range, deformation vibrations in the carbon skeleton, and vibrations with a change in the dihedral angle in the 300–750 cm^−1^ range [56,58,59].

### 2.2. FTIR Spectroscopy Study

**Figure 2:**
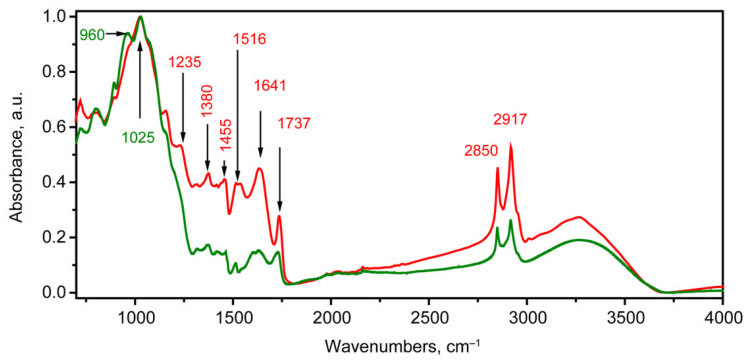
FTIR spectra of the grains infected for 40 days (red) and healthy grains (green).

Considering the IR absorption spectra of infected and healthy grains, a number of significant differences were noted (Figure 2), namely, a relatively large absorption in the region of stretching hydrogen vibrations in C-H bonds, as manifested by a large absorption for the peaks of 2850 and 2917 cm^−1^ against the baseline background after normalization in the range [0, 1]. In the region below 1800 cm^−1^, the greatest difference with this type of normalization was carried out for the 1330–1750 cm^−1^ range. In the high-frequency side of this range, a large absorption was noted for stretching vibrations in C=O bonds with a frequency in the 1725–1740 cm^−1^ range [60]. In addition, for the infected sample (Figure 2, red curve), a much higher absorption and a clearly expressed character were observed for the typical protein peaks: Amide I (1641 cm^−1^), Amide II (1516 cm^−1^), and Amide III (1235 cm^−1^). Also, a higher absorption was observed in the 1370–1460 cm^−1^ range. In the high-frequency part of this range, the greater relative contribution of deformation vibrations in the methylene groups (δ(HCH)) correlated with the greater relative contribution in the region of stretching vibrations (range 2800–3000 cm^−1^). And, in the low-frequency part, hydrogen deformation vibrations in the CCH groups and deformation vibrations in the CH_2_ and CH_3_ groups conjugated with others were observed [56]. It should be noted that in the region of stretching vibrations for healthy grains, the presence of two pronounced peaks with a maximum near 1025 cm^−1^ (the main one) and a weaker one at 965 cm^−1^ was observed, which were interpreted as stretching vibrations in the C-O and C-C bonds in various polysaccharides, including the antisymmetric stretching vibration in C-O-C and β(1-4)-glycosidic linkage.

In summary, the conducted studies of the healthy and infected grains composition made it possible to confirm the presence or absence of contamination using non-destructive optical methods and to make a control selection for a subsequent study of the possibility of separating samples using the hyperspectral imaging method.

### 2.3. Hyperspectral Imaging Study

In order to determine the criteria for differentiating healthy and infected grains, a study of the reflectance properties was carried out using the hyperspectral imaging method. Despite its similarity to a laboratory method such as the reflection spectroscopy technique, hyperspectral imaging has a number of specific features associated with conducting measurements under real conditions. The possible non-static behavior of the object requires a reduction in the imaging time for conducting quasi-static measurements. This leads to a decrease in the exposure time in each spectral band, due to which the sensitivity of the camera become significant depending on the wavelength and the dark current. Another specific condition is the possible presence of a background radiation contribution to the investigated spectral range—primarily natural solar radiation at the Earth’s surface taking into account atmospheric absorption, as well as artificially created by lighting devices and heated objects. The latter can be significant in the middle and near IR regions, which, together with the low sensitivity of the silicon based detector in the near IR region and short exposure times, leads to significant noise from the long-wavelength limit of the range. The obtained result can also be affected by the orientation of the sample relative to the incident radiation due to both different orientations of the sample surface, i.e., a purely geometric aspect leading to less reflection in the direction of the radiation receiver, and qualitatively different light absorption properties from the different sides of the grain.

**Figure 3:**
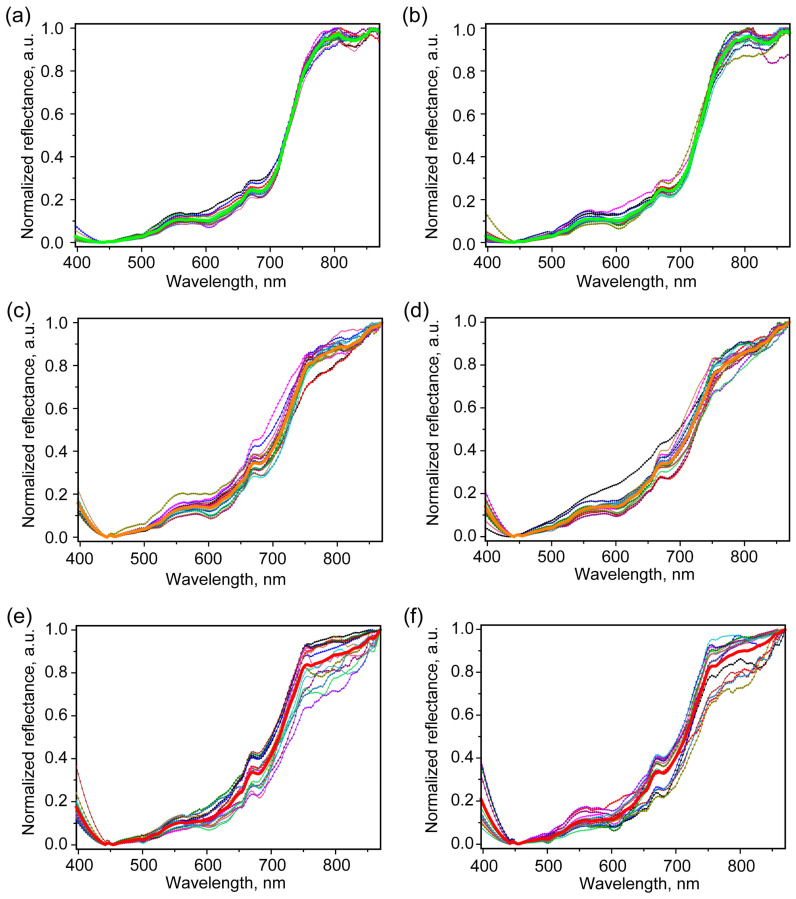
Normalized reflectance spectra of healthy grains from the top (**a**) and bottom (**b**) sides (lines with symbols); normalized reflectance spectra of grains infected for 5 days from the top (**c**) and bottom (**d**) sides (lines with symbols); and normalized reflectance spectra of grains infected for 40 days from the top (**e**) and bottom (**f**) sides (lines with symbols). The thick lines show the averaged spectra for different cases.

Reduced sensitivity at the range boundary, coupled with a short exposure time, can lead to a strong influence of noise on the subsequent chemometric analysis. In this regard, the original range recorded by the camera at 397–1000 nm was reduced to 397–870 nm, with subsequent smoothing using the Savitzky–Golay method [61]. Next, normalization [0, 1] was carried out in accordance with how it is described in Section 3.5. In this case, two types of probable variants of the grain orientation were considered: the first type was orientations closer to the back-up position and the second type was orientations closer to the groove-up position. Furthermore, these options were conventionally designated the top side case and bottom side case. Processed reflectance spectra in the 397–870 nm region for the cases of healthy grains, and grains infected for 5 and 40 days are demonstrated in Figure 3.

A number of common features can be identified in them. For both spectra, a sharp increase in reflectance in the range of 670–770 nm is noted, as well as in previous studies [27]. However, the course of this change is qualitatively different for healthy and infected grains. For healthy grains, less variability in the data in the presented range is noted. Among healthy grains in the top case, slightly less variability in the data in the region of maximum relative reflectance (>740 nm) can be noted. In both cases of healthy grain orientations, at least one extreme region is noted, and often two in the range of 740–870 nm.

At the same time, for the minimum reflection signal, a fairly flat course of the curve is noted in the range of 440–470 nm. It has been observed that the presence of a more pronounced minimum within this range is also noted in the case of infected grains. In the region of 400–550 nm, the reflectance minimum is about 450 nm. In general, the absorption in this region possibly could be due to the polycyclic aromatic compounds or compounds with multiple conjugated alternating double carbon bonds in the chains. As it was highlighted in [62], some of the *Fusarium* fungi, e.g., *F. graminearum*, may have a quite complex variety of the synthesized pigments. In *F. graminearum*, it was mentioned among the major synthesized pigments, such as the polycyclic pigments aurofusarin, rubofusarin, bostrycoidin and its various derivatives, as well as a number of carotenoid pigments with linear chain of double carbon bonds [62,63,64]. The former is mainly produced during specific light conditions [64] and among the most common are torulene and neurosporoxanthin. Although the characteristic absorption properties of carotenoids are quite sensitive to the pH values, their common feature is the broad absorption band with a maximum within 400–500 nm [65,66,67,68]. This range, coupled with the steeper rise in the reflectance spectrum for infected grains as they approach the short-wavelength boundary, suggests the possible presence of carotenoids.

However, the characteristic appearance of carotenoid pigments is yellow–orange and correlates with an earlier stage. Moreover, the rather flat course of the reflectance spectra from infected grains in the 500–550 nm range may indicate the presence of another type of pigment absorbing in this region, e.g., the polycyclic type pigments. As was mentioned earlier, *F. graminearum* can synthesize various polycyclic pigments [62] including bostrycoidin. Additionally, *F. poae* can also synthesize aurofusarin [63]. The only identified reference about *F. roseum* pigments was connected with the carmine pigment [69]. Bostrycoidin and its derivatives have absorbance bands in the 500–550 nm region, i.e., 528 nm (moreover, 488, 470, 318, 248, and 218 nm are attributed to bostrycoidin) [70]. Additionally, the blackish perithecial bostrycoidin and its derivatives, which are quite dark pigments with a dark violet hue, could be synthesized [62].

While the maximum reflection, starting from the early stage of infection, is mainly achieved closer to the boundary of the considered range, the reflectance maximum in the 780–810 nm region, which manifests itself in healthy grains, is absent in the ones after 5 and 40 days of infection. Starting from 740 nm, the reflection values in the spectrum of infected grains begin to increase monotonically along with the wavelength.

The observed spectral features (Figure 3) could be presumably interpreted as follows: due to the presence of fungi, an additional absorption peak about 780 nm can occur, attributed to the third overtone v(NH) [50,71,72]. Also, in the region next to the third overtone v(CH) in aromatic compounds, an absorption peak at 850 nm occurs [72]. Both the additional absorbance peaks for NH and aromatic CH bonds presumably deal with the presence of fungi, including metabolites and pigments. The abovementioned data correlate with the results of the FTIR and Raman spectroscopy studies in Section 2.2.

Considering the range of 470–700, a significant variation in the data (greater than in the case of healthy grains) is observed already for the 5-day infected grains. At the same time, due to the pigments produced by fungi that strongly absorb light, the value of reflectance in this range changes relatively little in comparison with the reflectance as it approaches the boundary of the range at 870 nm.

### 2.4. Hyperspectral Imaging Analysis

#### 2.4.1. Full Spectral Range

For the efficient and fast operation of the device, it is necessary to study the ranges and individual discrete wavelengths, of which reflectance is most variable and sensitive to the structure. Statistically valid reduction to these wavelengths using the PCA method will allow us to significantly reduce the total imaging procedure time.

**Figure 4:**
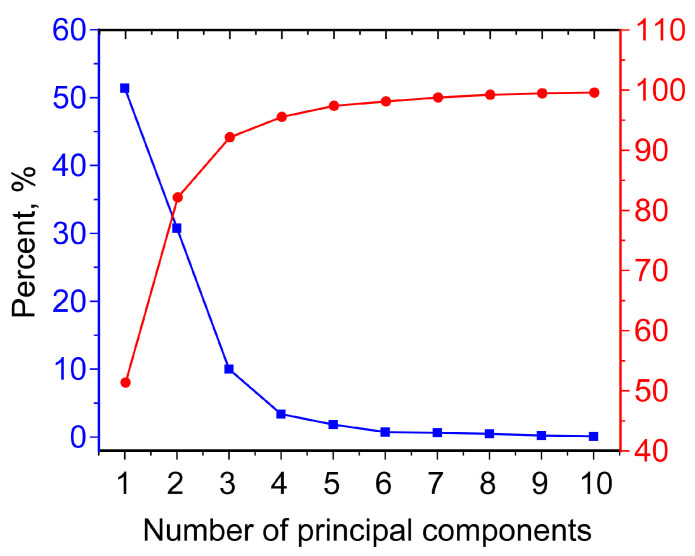
Percentage of variance from the principal component number (blue), as well as the total percentage of explained data (red).

At the first stage, spectral data were considered in the entire range of 397–870 nm. The dependence of the variance percentage on the PC number, as well as the total percentage of explained data are shown in Figure 4. PC1, PC2, and PC3, with this approach, explain approximately 51.4, 30.8, and 10.0% of the data, i.e., the first three PCs account for a total of 92.2% of the variance described. For PC4 and subsequent ones, the percentage of variance described is less than 3.5%.

**Figure 5:**
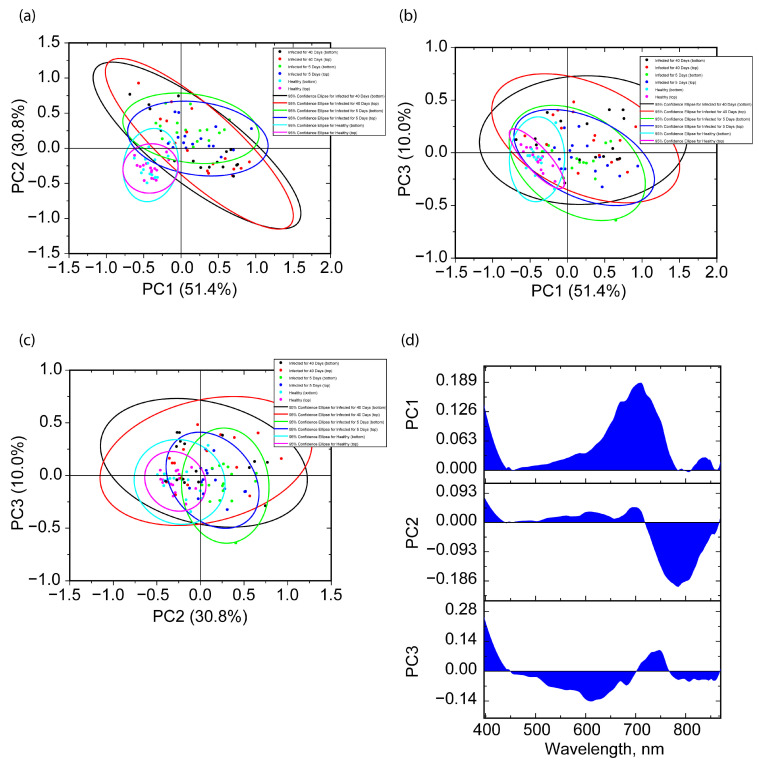
Score graphs for the first 3 PC pair combinations, (PC1, PC2) (**a**), (PC1, PC3) (**b**), and (PC2, PC3) (**c**), and the corresponding 95% probability ellipsoids, as well as the loading graphs for the first 3 PCs in the case of the full-range PCA (**d**).

Reflectance data, being constructed in new pairs of coordinates, are shown in Figure 5. As can be seen in Figure 5, the best separation of score values for healthy grains with top side case and bottom side case orientation occurs in the combination of PC1 and PC2. The greatest distance is achieved between the centers of the 95% probability ellipsoids on one side of healthy grains, and on the other side, the centers of the 95% probability ellipsoids for infected seeds, regardless of their orientation. In the case of the graphs in Figure 5b and Figure 5c, data separation is achieved due to PC1 or PC2, respectively. PC3 does not significantly contribute to separation, despite the more localized nature of the scores for healthy seeds in the top and bottom cases. Thus, this allows us to abstract from PC3 and further consider only the scores for PC1 and PC2 in the full region analysis.

In Figure 5a, one can note the peculiarities of the localization of the data sample and the shape of the ellipsoids of 95% probability. As mentioned above, for healthy grains, the localization of the score values is mainly within the third quadrant, i.e., when both PCs have negative values. At the same time, for healthy top cases, the shape of the ellipsoid of 95% probability is close to a circle, which means approximately the same distribution of the score values along the directions of PC1 and PC2. In the matter of the bottom case, an elongation of the ellipsoid is noted that is almost parallel to PC2. The loading graphs for PC1 and PC2 are shown in Figure 5d. For the graph of the PC1 loading, one can note the region of the largest weights located in the wavelength range of 738–833 nm, while the weights that are localized in the interval >90% by modulus from the maximum are in the interval of 765–806 nm. For PC2, two ranges can be distinguished, where comparable maximum values of the weights are achieved. One of them is located at the short-wavelength edge of about 400 nm. For the other range, the largest weights are in the 661–739 nm region, with weights that make up >90% of the maximum in the 690–717 nm range.

In the case of infected grains, much larger areas of 95% probability ellipsoids are noted. Their centers are in the area of small positive values in the first quadrant. In this case, the area of 95% probability ellipsoids for two different orientations on the 40th day is larger than similar ones on the 5th day and for healthy ones, which correlates with a larger variation in the reflectance values. For the values on the 40th day for the two orientations in the coordinates (PC1 and PC2), an extension of 95% probability ellipsoids along the direction PC2 = −PC1 is noted.

In general, the score values for the top and bottom cases are paired between each other for all three cases and are localized in approximately the same range, which indicates the possibility of separating healthy and infected grains at different stages, regardless of their orientation.

#### 2.4.2. Chemometric Analysis via Selected Spectral Bands

**Figure 6:**
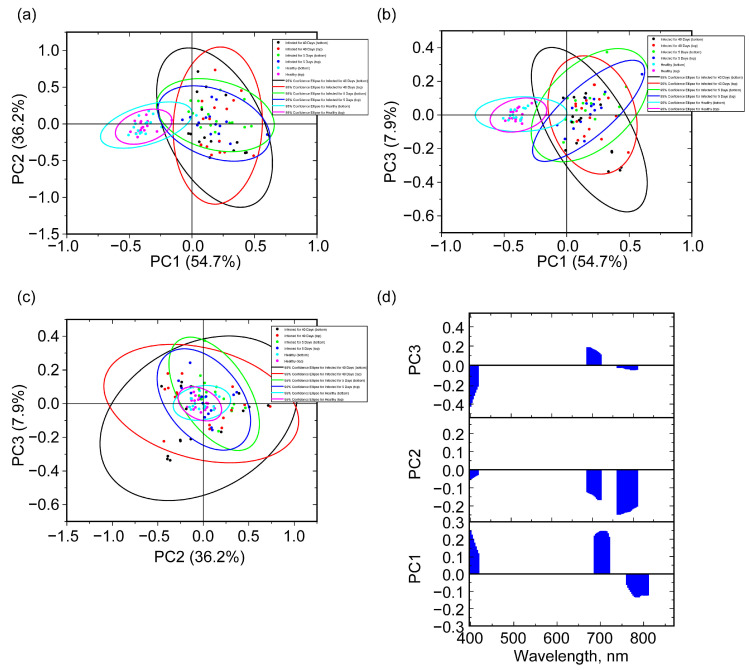
Score graphs for the first 3 PC pair combinations, (PC1, PC2) (**a**), (PC1, PC3) (**b**), and (PC2, PC3) (**c**), and the corresponding 95% probability ellipsoids, as well as loading graphs for the first 3 PCs in the case of selected bands in the PCA (**d**).

The speed of an infection analysis can be increased by reducing the entire data volume to certain spectral regions. In this work, we propose to select three regions for which the highest weights are locally noted in Figure 5d: 397–415, 690–717, and 765–806 nm. In this case, the spectral bandwidth of each band remains the same as in the case of the full range. The application of PCA to such data is demonstrated in Figure 6. With this approach, increased value of data variability explained by the first two PCs (54.7% and 36.2% for PC1 and PC2, respectively) is obtained in comparison with the full-range results. However, it should be noted that effective separation is carried out to a greater extent by PC1 than by the combination of PC1 and PC2, as in the case of the full range. This may be due to the fact that in the case of selected bands in the PCA for PC1, significant weights are observed at about 400 nm and 707 nm, which are more similar to PC2 for the full range (Figure 5d and Figure 6d). While in PC1, 760–780 nm is noted, which is more similar to PC1 for the full range (Figure 5d and Figure 6d). Although the counting data along PC2 and PC3 are localized quite well, the main contribution to the separation based on the selected band regions is made by PC1. As in the case of the full range, similar details with 95% probability ellipsoids are noted. Considering healthy grains, the ellipsoid for the top side has a smaller area compared to the bottom side. When it comes to 5 and 40 days of infection, the areas of the ellipsoids in the PC1 and PC2 coordinates are comparable for the top and bottom cases. In this instance, the difference in the coordinates of PC1 and PC3 becomes greater, but the percentage of explained information for PC3 is less (7.4%), with the localization of the largest weights around 400 nm. In the coordinates of PC1 and PC2 for the 5-day top and bottom cases, the areas of the ellipsoids are less than in the 40-day case.

#### 2.4.3. Chemometric Analysis via Selected Narrow Band Data

A further development of the idea of data dimensionality reduction can be the control of reflection values in certain narrow spectral bands. In practice, this can be implemented by irradiating the sample with LED radiation of a certain wavelength and detecting the reflected radiation at this wavelength. This approach is in fact a development of the RGB approach, only with the quasimonochromatic radiation sources, detectors, and spectral ranges of the study optimized for a specific task.

The selection of sensitive bands can be carried out on the basis of the results obtained in Section 2.4.1 and Section 2.4.2. It should be noted that, in general, due to the different values of reflection and the peculiarities of grain orientation, as well as the background signal that can be significant, the analyzing device needs to have data on the reflectance not only at the structure-sensitive wavelengths but also in the regions of maximum and minimum reflectance. The following set was chosen as the feature wavelengths: 400, 451, 708, 783, 801, and 863 nm. Moreover, the value of 451 nm was selected as the minimum value, which was noted in the overwhelming majority of spectra. At the same time, as written above, for healthy grains in the region of more than 780 nm, two comparable local maxima were found, while for infected grains an almost monotonous rise was noted (Figure 3). In this regard, for a reliable determination of the maximum in the reflection spectra, two values of 801 and 863 nm were taken, where, in practice, the maximum value can be selected and, together with the minimum value for the wavelength of 451 nm, reflectance normalization can be carried out for all the other wavelengths according to Formula (1).

**Figure 7:**
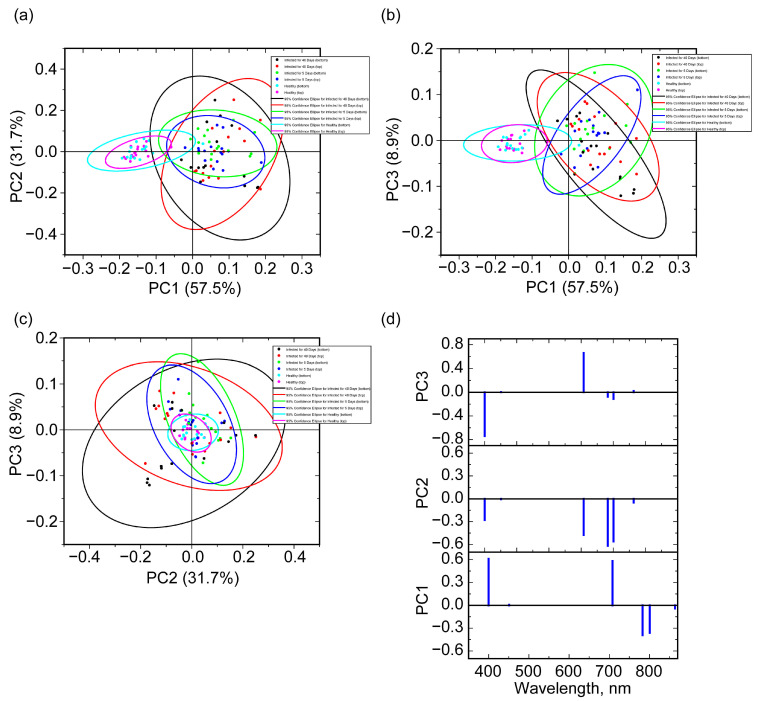
Scores graphs for the first 3 PC pair combinations, (PC1, PC2) (**a**), (PC1, PC3) (**b**), and (PC2, PC3) (**c**), and the corresponding 95% probability ellipsoids, as well as loading graphs for the first 3 PCs in the case of a selected set of feature wavelengths (**d**).

The above choice of maximum and minimum values is reflected in the loading graphs for the first three PCs, which account for 98.1% of the explained data variability (Figure 7). The weights for the wavelengths of 451 and 863 nm are small, while for the wavelength at 801 nm the weights of PC1 and PC2 are significant, which is associated with a much stronger variability in reflection for infected grains, regardless of the orientation. At the same time, by analogy with the results in Section 2.4.2, the separation also mainly occurs by PC1 with the largest weights at 400 and 708 nm. In the coordinates of PC2 and PC3 for the scores of healthy grains, a large localization is noted near zero values, while the scores values for infected grains are much more strongly delocalized and the areas of their 95% probability ellipsoids are significantly larger than for healthy ones. Moreover, the ellipsoids for the 40-day infected grains are larger than the analogs for 5-day infected grains, regardless of the grain orientation.

Thus, within the framework of this approach, it is proposed to track the processed reflectance at the 400, 708, 783, and 801 nm wavelengths using 451, 801, and 863 nm as auxiliary ones for proper reflectance normalization. To assess the predictive ability of the model, linear discriminant analysis (LDA) was performed using scores for the first three PCs. In this case, all 64 infected samples were classified correctly. At the same time, one healthy grain was erroneously classified as infected, and the remaining 31 grains were classified correctly. Thus, the total accuracy was 0.989, the sensitivity was 1.00, and the specificity was 0.969. In the current study, the entire dataset was used. A similar value was obtained for the full range of data, and in the case of the usage of selected spectral bands, allowing us to hope for a high predictive ability of this model. A number of works summarized in a review [6], based on the consideration of certain model systems and methods of data processing and analysis, also proposed feature wavelength sets that allow us to separate healthy and *Fusarium*-infected grains with a high degree of success. When comparing the obtained results, it is necessary to take into account, first of all, the parameters of the model samples that were used to conduct the study: what type of fungi was used for infection, what the features were during the growth of the fungus, and what the duration of infection was (or at least the degree of infection). The procedure for obtaining data and their subsequent processing are also important. Among the works carried out on similar topics, the following can be distinguished with comparable results [27,47,51]. The advantage of this work is the use of two selected infection times by duration. In addition, the advantages include a more reliable normalization procedure in the field compared to [27,47,51], where normalization with a dark current and a white reference was used. The implementation of the approach with the selected wavelengths allows one to move away from the use of complex-to-implement hyperspectral cameras to cheaper, more specific, and, due to the need for less data processing, faster systems. All of these can contribute to the development of flow systems and/or systems for analyzing large amounts of data with a relatively low cost due to cheaper optical components. The disadvantages include a more complex mechanism of the disease and the formation of metabolites, which require additional research depending on the temperature, humidity, and light conditions of growth, as well as the specificity of the equipment: detectors and lighting diodes. The restructuring of this equipment under changed conditions may require additional material costs and time. In this regard, research into the methods necessary for the effective detection of infected seeds under various conditions continues.

In [27,47,51], PCA was also used for data preprocessing, as in the present study, as one of the cost-effective dimensional reduction techniques. In [27,47], wheat grain samples with different degrees of infestation were studied, while in [51], visible signs of infestation were noted. At the same time, the proposed study allows us to further use the obtained spectral criteria as the basis for detection methods simultaneously at both early and late stages, simultaneously on the 5th and 40th days of infection, i.e., covering a fairly wide defined time range of the infection duration. As a result, in [51], a high degree of correct classification was noted when using data for wavelengths of 550 and 710 nm. In this study, the spectral band with 708 nm wavelength was noted to be important in various datasets. At the same time, the wavelength of 550 nm was considered far from the most important in this study, which may be associated with a relatively low reflectance and data variability at this wavelength for the studied samples. In general, it should be noted that for various fusarium pigments with a developed system of conjugated double bonds of a linear or polycyclic nature, strong absorption may be present in this region. In addition, grains with a high degree of contamination can have a rather dark shade, which differs in absolute value from reflectance at a similar wavelength. However, normalization of the type [0, 1] can significantly affect the absolute value. In [47], six spectral bands are proposed, where four wavelengths located in the studied range of 397–870 nm in this work: 484, 567, 684, and 817 nm. The band at 567 nm is close to 550 nm, as discussed earlier. In turn, 684 and 817 nm are close to 708 and 801 nm considered in this work, but differences can occur, including due to the specific contamination in this work, caused by several species of fungi. In [27], with data preprocessing using PCA, seven wavelengths were chosen: 491, 528, 652, 678, 686, 690, and 764 nm. A comparison of the results presented in all three works [27,47,51], as well as the results from this work, shows that the most sensitive wavelengths often are in the range of sharp changes in reflection of 670–770 nm, where, on the one hand, fairly large reflection values are noted for different types of normalization, and, on the other hand, significant variability is noted for contaminated grains. In this study, there are also selected wavelengths that are quite close to this region at 708 and 783 nm.

Summarizing the choice of narrow band data (400, 451, 708, 783, 801, and 863 nm) together with the discussion in Section 2.3 regarding the features in the reflectance spectra, an interpretation and explanation for the predisposition to choose these wavelengths could be offered as follows: the wavelengths of 400 and 451 nm are responsible for the changes in the absorption spectra associated with the pigment composition. And the predisposition to choose these wavelengths is associated with the production of pigments. The wavelengths of 783, 801, and 863 nm are also presumably associated with structural features and the resulting bonds, including the overtones, which are more pronounced in NIR for contaminated samples. It should be noted that in addition to the change in reflection compared to the uninfected case due to absorption on overtones, it is also necessary to take into account the possible change in the refractive index of the infected medium at different stages of infection, which has a very complex behavior. Moreover, some contribution to the change in reflection can also be made by a change in the shape of the surface. In the short-wavelength part, where absorption from pigments is significant, it should be taken into account that the pigment composition produced by *Fusarium* fungi can vary. In this regard, for various subsequent combinations of fungi and individual varieties of fungi considered in further works, a general idea can be proposed, which consists of choosing a combination of two ranges and an additional selected wavelength for normalization. Wavelengths from the first range refer to the wavelengths at which the greatest and least absorption of the fungal pigment is observed in the region of 400–600 nm. Wavelengths from the second range refer to the spectral regions where absorption of overtones is noted, for hydrogen bonds more characteristic for the protein medium, for example N-H and = C_aromatic_-H.

## 3. Materials and Methods

### 3.1. Grain Selection

The grain materials of the Timiryazevka 150 wheat variety, harvested in 2024, were selected for this research [73]. Grain crops in this study were grown in the Central Black Earth zone of Russia, and samples were taken from agricultural fields of the Federal Scientific Agroengineering Center VIM in the region with coordinates 46.121373 N°, 41.496803 E°. The weight of 1000 grains of wheat of the studied variety is within 38–47 g. The grain contains 9.8–10.4% proteins, and the percentage of gluten is 24.6–27.8%. The variety of wheat under study demonstrates resistance to brown rust, yellow rust, stem rust, and powdery mildew. The average yield of the Timiryazevka 150 variety over three years is 120 centners per hectare. For the hyperspectral imaging study, 100 grains were selected for healthy samples and 100 grains for subsequent infection.

### 3.2. Infected Grain Preparation and Selection

Infected grains were detected by the testing laboratory of the Russian Agricultural Center, Rostov Region Branch, registration number ROSS RU 0001.21PT12. The analysis was conducted in accordance with internal state standard GOST 31646-2012 [24]. The full procedure was performed in similar way as in [24] for 2.0 ± 0.1 kg of wheat grain. This method was applied in order to analyze the surface and the shape of the grain by the color of the embryo, the structure of the endosperm, and the presence of fungi. Infected grains were shrunken and had spots on the surface. The structure of the endosperm was loose and its vitreousness was lower compared to healthy grains. The color of the cut embryo was dark brown. At the same time, a light gray fungal coating was located on a part of the embryo. According to laboratory studies, it was established that 30 ± 2% of the 2.0 ± 0.1 kg of wheat grain was infected with *Fusarium* genus fungi, among which the following fungi were identified by morphological properties: *Fusarium graminearum* Schwabe FG-30, *Fusarium poae* Kr-20-14, and *Fusarium roseum (sambucinum)* St-20-3.

The procedure for isolating the aforementioned fungal micromycetes was the following: the contaminated biomaterial was washed with running water, air-dried, and chopped with scissors into 2–4 mm long fragments. Then, using pincers, each fragment was dipped in 96% ethanol for 3 s, flamed, and placed on the surface of potato–glucose agar with gentamicin in standard glass Petri dishes (100 fragments, 4 fragments per dish). The specimens were incubated in a thermostatted incubator at 26–28 °C for 7–10 days (until colonies appeared), and then the colonies were transferred to a fresh nutrient medium (potato–glucose agar), grown, and micro specimens were prepared. Finally, samples were viewed under a microscope and the taxonomic affiliation of the microorganisms was determined. Before the infection with pathogens, parts of the mycelium of the same size (sectors of ⅛ of the dish) were selected using a disposable scraper and transferred to separate sterile vials. Then, 5 mL of distilled water was added to the vials using a Pasteur pipette. The resulting solution was shaken in an ultrasonic bath for 3 min for better saturation with pathogen spores. Using separate Pasteur pipettes, 1 mL of the washings from each vial was transferred to Petri dishes with grains, and the dishes were sealed with parafilm. After all stages, all Petri dishes were packed in airtight bags and stored in a thermostatted Jiupo 500 L Plant Growth Chamber at a 28 °C temperature.

A mixture of *Fusarium* genus fungi (*Fusarium graminearum* Schwabe FG-30, *Fusarium poae* Kr-20-14, and *Fusarium roseum (sambucinum)* St-20-3) was isolated from the identified infected grains. The mixture was plated on growth medium and grown under standard conditions. After that, a controlled seed infection procedure was performed.

**Figure 8:**
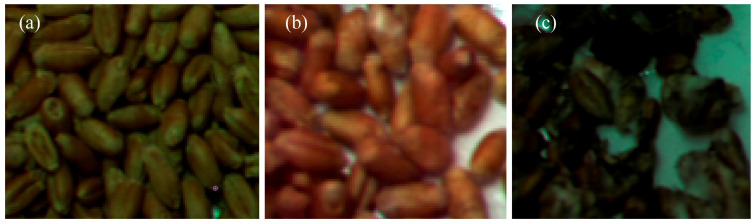
Comparative photographs of healthy grains (**a**) and grains after 5 (**b**) and 40 (**c**) days of infection.

In order to conduct this research, grains were selected on the 5th and 40th days of infection; these cases will be further considered as variants of weak and strong infection (Figure 8).

### 3.3. Spectroscopic Investigations

In order to confirm the infection of grains, investigations with Raman spectroscopy and FTIR spectroscopy were performed. The main focus was placed on the application of the hyperspectral imaging for grain infection detection as a technique that allows us to perform a rather quick analysis of the large amount of data in noncontact way with needed accuracy. Taking into account these features, the Raman spectroscopy as well as FTIR spectroscopy results are considered as additional ones.

#### 3.3.1. Raman Spectroscopy

The Raman spectra were obtained in the backscattering geometry by the confocal Raman spectrometer SENTERRA II (Bruker, Germany). The excitation of Raman scattering was performed with a 785 nm laser. In order to avoid sample degradation, the low laser power density was set. The actual laser power was about 0.1 mW under a 20× objective with a numerical aperture (N.A.) equal to 0.4. The accumulation time was 100–180 s, with two repetitions. The diffraction grating was 400 g/mm. The spectra were recorded in the region of 80–3700 cm^−1^. The aperture was 25 × 1000 μm. Due to the locality of the acquired signal the spectra were obtained from the three points of the grain from the both sides and compared with the corresponding results studied earlier [53,54,55,56,57,58,59,60]. In order to achieve better clarity, the fluorescence background was subtracted and the standard [0, 1] normalization procedure was performed via OriginPro2021b software (OriginLab Co., Northampton, MA, USA).

#### 3.3.2. FTIR Spectroscopy

The FTIR absorbance spectra were obtained by an FTIR spectrometer Nicolet 6700 (Thermo Fisher Scientific, Waltham, MA, USA) equipped with a liquid nitrogen-cooled MCT-A detector. The ATR accessory with a diamond crystal was used. The beamsplitter was XT-KBr. The light source was a Globar (SiC) lamp. The spectra were obtained in the 650–4000 cm^−1^ region with a 4 cm^−1^ resolution. The number of performed scans was 80. The apodization function was Blackman–Harris [74], and the Mertz phase correction was applied [75]. The obtained spectra were normalized according to the standard [0, 1] normalization procedure using the OriginPro2021b software (OriginLab Co., Northampton, MA, USA).

### 3.4. Hyperspectral Imaging Procedure

**Figure 9:**
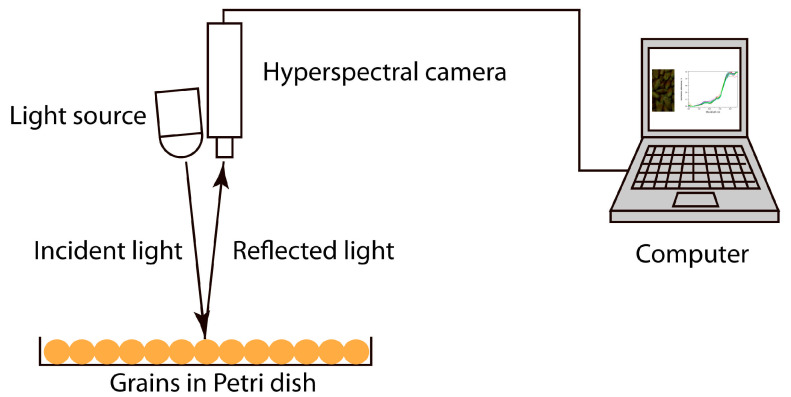
Schematic representation of the setup for taking samples, indicating the main elements.

For the hyperspectral imaging procedure, 100 healthy grains, 100 grains contaminated for 5 days, and 100 grains contaminated for 40 days were selected. They were distributed randomly on a Petri dish for a subsequent study of the effect of ordering. A schematic representation of the imaging setup is shown in Figure 9. The working distance from the camera to the plane with the grains was 154 mm.

Hyperspectral data were obtained using a portable hyperspectral camera Specim IQ. The spectral range of reflectance spectrum was from 397 to 1000 nm, which corresponds to the visible and partially near IR region (hereinafter referred to as VNIR). The device contains a CMOS detector. The number of spectral bands where reflection was obtained was 204. At the same time, each spectral band had an average spectral range of 3 nm. The image in the focal plane was recorded with a spatial resolution of 512 pixels for each dimension, which allowed us to compare the spectral analysis result with individual areas on the grains, taking into account their different orientations. The camera is 207 mm wide, 91 mm high, and 74 mm deep, and when equipped with a lens, its total length reaches 125.5 mm. The device weighs 1.3 kg, which makes it easy to carry and use both in the field and in stationary installations, including for aerial photography.

In this work, the 870–1000 nm range was excluded from further processing due to the gradually decreasing sensitivity of the silicon detector as the long-wavelength boundary close to the band gap of silicon was reached and relatively weak changes in the reflectance spectrum were observed. Thus, for further data processing, the 397–870 nm region with 159 spectral bands per spectrum was chosen. To study the effect of orientation-dependent reflectance, 16 randomly oriented grains were selected for each group of states with various orientations, i.e., healthy samples and samples at 5 and 40 days of infection, predominantly from the back side (smooth side identified by the absence of a groove further called the top side case), and healthy samples and samples at 5 and 40 days of infection, predominantly from the grooved side of the grain (these grains were identified by an asymmetrically located groove further called the bottom side case), i.e., 48 grains from the back side and 48 grains from the grooved side, therefore, 96 grains in total. The spectrum from each grain itself was obtained by averaging 4 reflectance spectra from 4 corresponding pixels falling on this grain.

### 3.5. Data Preprocessing Procedure

The process of preparing the data for the chemometric analysis consisted of two parts. In the first stage, smoothing was performed using the Savitzky–Golay method. The following parameters were selected: a second-degree polynomial and 25-point spectral windows.

In the second stage, the obtained spectra were normalized. In this part, due to different reflections from the arbitrarily oriented surfaces of wheat grains, as well as a potentially significant background signal, the data processing procedure is important. Existing hyperspectral imaging data normalizations can be divided into both data normalizations for a specific wavelength for the entire image area and normalizations of the entire spectrum in a specific image area [76]. In this work, the approach that involves normalizing the entire spectrum in a specific pixel of the image was chosen. Here, unlike previous works using normalization of the type (R(λ) − R_dark noise_(λ))/(_Rwhite reference_(λ) − R_dark noise_(λ)), where R(λ) is the sample reflectance, R_dark noise_(λ) is the dark noise level of the signal, and R_white reference_(λ) is the reflectance from the white reference sample at the wavelength λ [27,47], it was decided to perform normalization based on the shape of the reflectance spectrum. It was assumed that this would allow us to bypass a number of technical difficulties in practice, for example, extraneous scattered light can lead to an additional contribution to the reflection. The feature of [0, 1] normalization can be especially useful given that the HSI area may be subject to random illumination by reflections from a highly reflective surface or emissive sources, e.g., external point light sources, leading to a spatially non-uniform shift in the reflection value, i.e., the illuminated area may have larger values. At the same time, the normal (non-illuminated) area surrounding it may have lower values. Thus, even at the same states, the additionally illuminated area and non-illuminated area will have different ratios of reflectance at selected wavelengths, which may influence decision-making. The [0, 1] normalization makes these criteria less susceptive to the random illuminations. This is especially critical for strongly absorbing spectral regions, for example, with a high degree of grain degradation during infection, when it becomes dark due to the presence of a high concentration of pigment. At the same time, due to the orientation of the grain at an angle relative to the imaging plane, less reflected light can enter the HSI spectrophotometer camera, which will lead to a lower value, including in the areas of maximum value. Considering the geometric factor of reflectance, the signal scaling procedure in such normalization can be both an advantage and a disadvantage. Together with a well-established preliminary smoothing procedure [0, 1], normalization can lead to a greater difference in values for infected and healthy grains. Another additional advantage, especially in field conditions, is the lack of need for calibration using a white sample and dark current (due to the inevitable influence of ambient lighting), but only the need to evaluate the sensitivity and response of pixels in the camera, which can be carried out under laboratory conditions.

In this regard, within the framework of this work, the normalization [0, 1] was chosen for the reflection values in the spectrum, i.e.,

R_norm_ = (R(λ) − R_min_)/(R_max_ − R_min_) (1)

where R(λ), R_min_, and R_max_ are the reflectance values at wavelength λ and the minimum and maximum values in the smoothed spectrum. The search for the minimum and maximum is performed for the smoothed spectrum, not the original one, in order to avoid the possible influence of noise. Such normalization allows changes in the band ratio in the dynamically important range of values of radiation reflected from the sample to be tracked. Data processing was performed using OriginPro2021b software (OriginLab Co., Northampton, MA, USA). In the article, unless explicitly stated, reflectance data already processed in the manner described above are considered.

#### Chemometric Approach Procedure

Due to the large dimensionality of the hypercube data, it was decided to use chemometric analysis methods to identify implicit dependencies. From a practical point of view, the computational cost of the analysis method plays an important role in the fastest decision-making in streaming systems with large amounts of data. One of the least labor-intensive but quite effective methods is the principal component analysis (PCA) method. It allows one to significantly reduce the data dimensions, as well as clearly demonstrate the most significant spectral ranges for decision-making, which is reflected in the largest weights on the loading graphs. This method makes a linear transition to new orthogonal variables, the so-called principal components (PCs) [77]. As a result of the analysis, spectral regions with the greatest impacts on the classification ability were identified. The PCs are ordered in descending hierarchy of variability, which made it possible to reduce the dimensionality of the spectral data to several components that accumulate a fairly large amount of information. In this work, it was decided to take into account the first three components, which accumulate >90% of the data variability.

In order to perform the maximum dimensional reduction, the obtained score values for first 3 PCs were used to classify healthy and infected grains using linear discriminant analysis (LDA), which is a generalized case of Fisher’s linear discriminant method [78]. The PCA-LDA approach is a rather simple but fairly reliable classification method that is relatively uncomplicated to implement in practice and allows for fairly the quick and easy mathematical classification of massive data. The predictive accuracy of the resulting model was assessed based on parameters such as accuracy, sensitivity, and specificity, which are defined as follows:

Accuracy = n_correct_/n_total_ (2)

Sensitivity = TP/(TP + FN)(3)

Specificity = TN/(TN + FP)(4)

where n_correct_ and n_total_ are the number of samples classified in the correct way and total number of samples, respectively; TP (true positive), FN (false negative), TN (true negative), and FP (false positive) are the number of positive samples classified as positive, the number of positive samples classified as negative, the number of negative samples classified as negative, and the number of negative samples classified as positive. In other words, FN means a positive result that corresponded to the infected samples and FP means a negative result ascribed to healthy grains.

The complete dataset consisted of 16 healthy grains, oriented predominantly from the top side case; 16 healthy grains, oriented predominantly from the bottom side case; 16 grains infected for 5 days, oriented predominantly from the top side case; 16 grains infected for 5 days, oriented predominantly from the bottom case; 16 grains infected for 40 days, oriented predominantly from the top case; and 16 grains infected for 40 days, oriented predominantly from the bottom case used for classification. In summary, the total number of grains used for statistical analysis was 96 items, where 32 items in total, regardless of orientation, were healthy and 64 items in total, regardless of orientation, were infected. Classification into healthy and infected grains and the estimated classification error were made based on the size ratio of healthy to infected sets of 1 to 2, i.e., with corresponding weights proportional to the sampling.

In this article, normalized VNIR spectra (see Section 3.5 for descriptions of processing) were analyzed via OriginPro2021b software (OriginLab Co., Northampton, MA, USA) and, in particular, using its built-in Principal Component Analysis for Spectroscopy v1.3 application.

## 4. Conclusions

The hyperspectral imaging approach in combination with chemometric methods was applied to study the possibility of separating healthy wheat grains and wheat grains infected with several species of *Fusarium* genus fungi, namely, the common *Fusarium graminearum* Schwabe FG-30, *Fusarium poae* Kr-20-14, and *Fusarium roseum (sambucinum)* St-20-3. Such contamination is closer to real field conditions. In addition, the objective of this study was to investigate the effect of the grain orientation on the classification possibility. This aspect is important in the conditions of grain classification, for example, located on a conveyor belt. In this regard, hyperspectral imaging of randomly ordered samples of healthy and infected wheat grains was performed. Within the framework of PCA, a study of spectral regions was carried out in order to determine the most sensitive wavelengths. In this case, smaller variability in the data was noted for healthy grains in the case of an orientation predominantly with the top side than in the case of the bottom side. For 5-day and 40-day infections, the difference in orientations was relatively small, while the positions of the scores shifted in the coordinates (PC1 and PC2), and the ellipsoids of 95% probability gradually increased with the time of infection, which indicated a greater variability in the data and allowed the effective separation of healthy and infected grains. The cases of reducing the dimensionality to three ranges (397–415, 690–717, and 765–806 nm), as well as to a set of individual spectral bands (400, 451, 708, 783, 801, and 863 nm) were also considered.

The wavelengths of 400 and 451 nm fall into the region where pigment absorption is present, and wavelengths of 783, 801, and 863 nm are in the region where there may be overtones absorption from hydrogen vibrations, including in N-H and = C_aromatic_-H bonds. Thus, this approach is based on the wavelength ranges characteristic for the fungal pigment and overtone spectral regions associated with the protein environment. The determined wavelengths were compared with similar research results obtained earlier. In these cases, PCA was still able to explain a fairly large percentage of the data variation (>90%) while maintaining a comparable data classification accuracy with the PCA-LDA approach. At the same time, the weights in PC1, which was mainly used to separate healthy and infected grains, changed qualitatively. Considering the individual spectral bands, it was found that the first three principal components allowed the classification of healthy and infected grains with a total accuracy of 0.989 with the PCA-LDA approach. The demonstrated approach based on sensitive wavelengths via relatively computationally inexpensive methods may have potential for further application to identify infected grains.

## Data Availability

Data may be provided if there is a reasonable request.
